# The Role of Motor Activity in Insight Problem Solving (the Case of the Nine-Dot Problem)

**DOI:** 10.3389/fpsyg.2019.00002

**Published:** 2019-01-23

**Authors:** Vladimir Spiridonov, Nikita Loginov, Ivan Ivanchei, Andrei V. Kurgansky

**Affiliations:** ^1^Laboratory for Cognitive Research, The Russian Presidential Academy of National Economy and Public Administration, Moscow, Russia; ^2^Laboratory for Cognitive Research, National Research University Higher School of Economics, Moscow, Russia; ^3^Laboratory for the Cognitive Psychology of Digital Interface Users, National Research University Higher School of Economics, Moscow, Russia; ^4^Laboratory of Neurophysiology of Cognitive Processes, Institute of Developmental Physiology, Russian Academy of Education, Moscow, Russia

**Keywords:** problem solving, insight, nine-dot problem, motor planning, preliminary motor training

## Abstract

Attempts to estimate the contribution made by motor activity to insight problem solving is hindered by a lack of detailed description of motor behavior. The goal of this study was to develop and put to the test a novel method for studying the dynamics of insight problem solving based on a quantitative analysis of ongoing motor activity. As a proper problem model, we chose the nine-dot problem (Maier, 1930), in which solvers had to draw a sequence of connected line segments. Instead of using the traditional pen-and-paper way of solving the nine-dot problem we asked participants to use their index finger to draw line segments on the surface of a tablet computer. We are arguing that successful studying of the role of motor activity during problem solving requires the distinction between its instrumental and functional role. We considered the functional role on the motor activity as closely related to the on-line mode of motor planning. The goal of Experiment 1 was to explore the potential power of the method and, at the same time, to assay the patterns of motor activity related to on-line and off-line modes of motor planning. Experiments 2 and 3 were designed to uncover the potential impact of preliminary motor training on the motor output of successful and unsuccessful problem solvers. In these experiments, we tested hypotheses on how preliminary motor training, which presumably played a functional role in Experiment 2 and an instrumental role in Experiment 3, affects the motor activity of a problem solver and hence their effectiveness in solving the problem. The three experiments showed consistent results. They suggest that successful solving of the nine-dot problem relies upon the functional role of motor activity and requires both off-line and on-line modes of motor planning, with the latter helping to overcome the perceptual constraints imposed by a spatial arrangement of the nine dots. The method that we applied allows for systematic comparison between successful and unsuccessful problem solvers based on the quantitative parameters of their motor activity. Through it, we found new specific patterns of motor activity that differentiate successful and unsuccessful solvers.

## Introduction

The concept of insight has remained in focus of researchers since its introduction in 1917 by Köhler (1921). An insight can be defined as the moment of sudden comprehension of a problem solution often accompanied by an aha experience (Öllinger et al., 2013, 2017). Since then, a considerable number of theoretical models have been suggested to explain insight (insight solution) in terms of various mental mechanisms: for example, heuristic search (Kaplan and Simon, 1990; Ormerod et al., 2002) or representational change (Ohlsson, 1984; Knoblich et al., 1999; Öllinger et al., 2013).

The most popular theoretical models usually do not consider the solver’s own motor activities which emerge while solving insight problems as a factor contributing to their solutions (Ohlsson, 1984; Kaplan and Simon, 1990; Knoblich et al., 1999; Ormerod et al., 2002). At odds with this view, data accumulated through a number of studies have shown that the motor activity of the solver is intimately woven into the fabric of the solving process. The solving process can be speeded up or delayed if preceded (Weisberg and Alba, 1981; Lung and Dominowski, 1985; Kershaw and Ohlsson, 2004) or accompanied (Thomas and Lleras, 2009) by the motor activity of the solver. The solver’s movements can even play a decisive role in choosing among possible solutions of the problem at hands (Werner and Raab, 2013). In the study by Werner and Raab (2013), participants were asked to solve a modified version of the Maier’s two-string problem. This version of the problem has two possible solutions: participants can either turn one of the strings into a pendulum by securing a weight to it (swing-like solution) or gain a higher position by stepping on the desk and connect the strings (step-like solution). Two groups of solvers participated in the experiment (Werner and Raab, 2013, Experiment 1). Prior to the test session, participants belonging to the first group were asked to swing their arms back and forth, while participants belonging to the second group had to step up onto and down off a chair. This experiment showed that participants from the 1st group more frequently chose the swing-like solution, while participants from the 2nd group preferred step-like solution. These and similar results are clearly not in line with existing models of insight and beg for an explanation.

Any attempt to estimate the contribution made by overt motor activity to a person’s success (or failure) in finding an insight problem solution is hindered by the lack of variables quantifying motor behavior. A common practice among researchers is to use variables such as the number of trials along with the overall time needed to solve the problem and the percentage of correct responses. Unfortunately, using these variables results in averaging out any potential temporal dynamics in ongoing motor activity and, therefore, brings about an inability to differentiate between successful and unsuccessful problem solvers based on the patterns of those dynamics.

**FIGURE 1:**
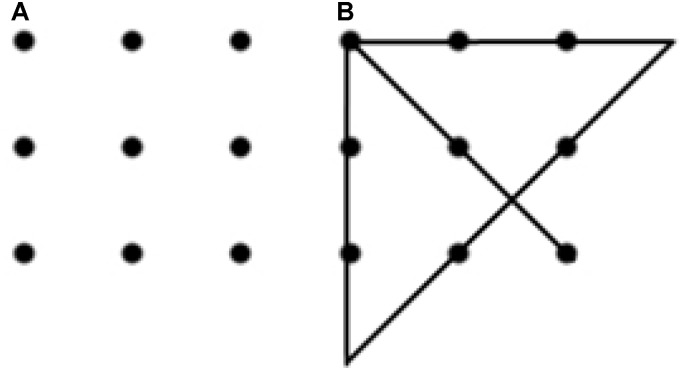
Maier’s nine-dot problem **(A)** and one of the possible solutions to this problem **(B)**. Participants are asked to connect the nine dots with four straight lines without taking the pencil off the paper (Maier, 1930).

In this work, our first priority was to develop and put to the test a novel method for studying the dynamics of insight problem solving based on a quantitative analysis of ongoing motor activity. As a proper problem model, we chose one of the most studied insight problems, the nine-dot problem (Maier, 1930) (see Figure 1A). This problem is traditionally considered insightful because it provokes the emergence of an inadequate initial representation, which hinders the solution: in the initial stages, the subjects connect dots with lines, without going beyond the limits of the square. To solve the problem, a radical change (restructuring) of the initial representation is required. It is this change of the initial representation, which is associated with insight (Scheerer, 1963). For a detailed analysis and criticism, see (Weisberg, 1995).

In the nine-dot problem, motor activity takes the form of sequential movements executed in order to draw a proper spatial trajectory – a sequence of connected line segments. Instead of using the traditional pen-and-paper way of solving the nine-dot problem we asked participants to use their index finger to draw line segments on the surface of a tablet computer. This allows for using variables that characterize the temporal structure of the graphical movements executed by problem solvers. Since the whole experiment is arranged as a block of trials (i.e., successive attempts to solve the problem), the sequence of parameters could be used to discover characteristic patterns of motor activity and to see if and how these patterns change across the series of trials.

Our second priority was to try to describe what patterns of motor activity distinguish between successful and unsuccessful nine-dot problem solvers.

## The Role of Motor Activity in Solving the Nine-Dot Problem

There are two roles that motor activity might play in solving insight problems: instrumental and functional. When taken in its instrumental role, the motor activity does not influence the nature of the solution but merely implements the solution already found with some other cognitive processes. For example, in case of the nine-dot problem, the instrumental role of motor activity would be limited by drawing a correct sequence of connected line segments (similar to the one shown in Figure 1B), which had been prepared in advance. The instrumental role of motor activity in solving other insight [e.g., 6-coin (Chronicle et al., 2004), 8-coin (Ormerod et al., 2002), 6 matches (Scheerer, 1963), etc.] and non-insight [e.g., 5 rings Tower of Hanoi (Anzai and Simon, 1979)] problems is also the implementation of the sequence of movements leading to the correct solution, which was previously constructed in the mind. The examples that illustrate the instrumental role of motor activities for relatively simple motor tasks are in: (Tessari and Rumiati, 2004; Tessari et al., 2006).

When playing a functional role, motor activity lays the very ground for the solution being sought, i.e., the motor activity directly affects the process of problem-solving and the outcome of that process. This view has received some experimental support (Grant and Spivey, 2003; Thomas and Lleras, 2009; Werner and Raab, 2013). Thus, in a study by Werner and Raab (2013), in experiment 2, the modified water-jar problem (Luchins, 1942) was used. This problem could be solved either by (1) subtracting the amount of water held by one of the smaller jars twice from the biggest one or (2) by adding the amount of water held by one smaller jar twice to the other smaller jar. As a prime for the subtraction solution (group 1), a 30-s preliminary procedure was used to move marble balls from the middle jar into two outer jars, while the priming for addition solution (group 2), a similar procedure of moving the same balls from the outer jars to the middle one. It was found that subjects of group 1 more often used the subtraction solution while their group 2 counterparts more often relied on the addition solution.

However, there are few such studies, and they are vulnerable to criticism. In most cases, it remains unknown whether the reported results are truly related to the functional role of motor activities rather than reflecting the form of some abstract idea hinted at by these activities. For example, in two similar studies (Thomas and Lleras, 2009; Werner and Raab, 2013) the arm swinging preceding the test session not only directly points to the movement pattern critical for solving the two-string problem but also indirectly prompts the abstract idea of a pendulum and similar ideas. Thus, experimental studies that have been conducted so far leave unanswered the question of how the motor activity relates to the process of solving insight problems. In particular, the question of whether motor activity plays a functional role also remains largely unanswered.

We assumed that in the case of the nine-dot problem, it may be related to a certain mode of motor planning. According to Wilson’s definition, two kinds of cognition have to be distinguished: “on-line” (or “situated”) cognition and “off-line” cognition (Wilson, 2002, p. 626). On-line cognition critically depends on the particular conditions (including spatial ones) in which they take place. It is linked to the properties of the surroundings and makes use of the latter in order to reduce the cognitive processing burden, is sensitive to different kinds of affordances which automatically trigger specific motor programs, etc. In contrast, off-line cognition takes place in the mental domain without any apparent influence of the surrounding environment.

The off-line vs. on-line distinction fully applies to a motor activity which includes two major phases, known as the motor preparation phase and the motor execution phase. It is often assumed that the most important cognitive processes take place during the first preparatory phase and that taken together constitute what is known as motor planning. In other words, the term “motor planning” refers to those cognitive processes that are related to a movement and precede it (Stanford, 2013).

One might think that motor planning is an off-line process by definition. However, studies of movements toward a spatial goal in the condition of the uncertainty of its position (Scott, 2012; Wong et al., 2015; Wong and Haith, 2017) and the data on the role of sensory feedback and its prediction (Scott et al., 2015) show that planning can be an on-line process. When relying on off-line planning, a problem solver prepares an entire movement sequence (or a substantial fraction thereof) ahead of time and then executes it uninterruptedly. In this mode, the only opportunity to estimate the surrounding environment and to select the appropriate movements is prior to the sequence execution. Similarly, the opportunity to estimate the results of the movement execution exists after the sequence has been executed. Therefore, one may say that off-line planning has a long but narrow horizon. In the case of the nine-dot problem, this mode of planning is akin to the notion of a “mental lookahead” (Ohlsson, 1984; MacGregor et al., 2001). Mental lookahead directs the heuristic search in the course of the problem solution due to the anticipation of new states within the “problem space.” Its range is limited (Ohlsson, 1984). In the course of solving the nine-dot problem, it can vary in horizon by representing from one to four straight lines (MacGregor et al., 2001). Regardless of the depth of the mental lookahead, the off-line planning is completed before any movement has occurred (drawing lines connecting dots).

In contrast, on-line planning goes hand by hand with movement execution. This mode of planning allows for a continuous re-evaluation of the surrounding conditions while taking into account the solution being searched for and the results of the already executed movements. Thus, when compared to off-line planning, on-line planning has a wide but short horizon. It opens different options to continue with the already started movement or movement sequence.

A major difficulty that the problem solver faces while attempting to find a solution to the nine-dot problem is incompleteness of the mental representation of the task, i.e., a lack of constituents (perceptual and abstract entities) which are critical for constructing a correct solution. Such incompleteness manifests itself in a limited repertoire of movements and results in an inability to solve the problem. An attempt to solve the problem usually begins with drawing straight lines along the outer sides of the nine-dot square which points to a rather narrow repertoire of movements.

**FIGURE 2:**
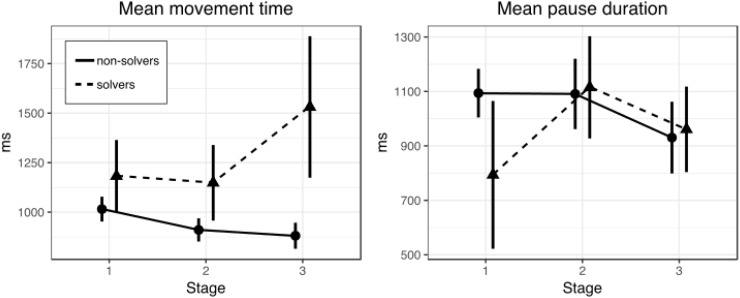
Mean movement **(left)** and pause **(right)** time in three stages of the nine-dot problem solving (Experiment 1). Bars represent within-subject 95% confidence intervals.

We assumed that in the case of the nine-dot problem, relying exclusively on off-line planning is insufficient in order to overcome this narrow repertoire of movements. In the study by MacGregor et al. (2001), a theoretical model was developed to explain heuristic search in the course of the solving of the nine-dot problem through the exploit of maximization and progress-monitoring heuristics with a variable lookahead depth ranging from 1 to 4 consecutive line segments. This model has gained empirical support from the experiments involving the problems similar to but way more simple than the nine-dot problem (MacGregor et al., 2001, Experiments 1, 2, 3). Thus, in experiment 1 of the cited paper, a percentage of participants who successfully solved the problems varied from 80 to 93% while no one solved the nine-dot problem. It seems that unlike the original nine-dot problem, the simplified problems (see Figure 2 in the cited paper) provide the stronger hints for the initial line segments which are the part of a correct solution. It helps solvers to rely on a shorter mental lookahead. However, the model does not explain the evolution of the line segments that are drawn by solvers. What begs for explanation is how the participants manage to go beyond the square area defined by the nine dots, i.e., to start and end the line segments outside this area. It is at this point that the on-line planning reveals its significant role.

The advantages of this mode of planning are as follows. First, within a single attempt to solve the problem (i.e., to draw a proper sequence of four connected line segments), on-line planning gives more opportunities to build a proper solution than does the off-line mode. This is because in the former case, the construction process goes on all the time, and it is not limited to the period of time prior to the sequence execution. Second, the evaluation of the intermediate results of movements makes it more probable to get an idea that a trajectory vertex (its joint or turning point) may not necessarily coincide with one of the nine visible dots. Finally, a permanent monitoring of motion, i.e., keeping track of an index fingertip position and its velocity, might bring into focus the idea of motion direction, whose spatial trajectory is a straight-line segment with off-dot margins. Under these circumstances, a problem solver may discover with a greater probability that a line segment does not necessarily begin or end with one of the visible dots and that the angle between two consecutive lines is not necessarily a right angle.

It is required by the nature of the nine-dot problem that the spatial trajectory (path) corresponding to its correct solution has to take a form of piecewise linear curve containing 4-line segments and connecting (passing through) all 9 points. However, these requirements do not impose any constraints on whether or not this trajectory is pre-planned as a whole ahead of its execution or on the timing of the fingertip movement along this path. The trajectories produced by solvers of the nine-dot problem showed multiple stops between positions of visible dots sometimes very long (up to several seconds). Because of that, we do not have any reason to think that off-line planning takes place during pauses in the spatial trajectory vertices. Instead, we made two assumptions. We assume that (A1) the off-line planning contribution is proportional to the average stop duration (inter-movement pause duration) and (A2) the contribution of on-line planning is proportional to the average movement duration (i.e., inversely proportional to the average movement velocity). These assumptions are supported by the following. First, longer movement sequence is characterized by a longer latency time and a longer execution time of its units (for a review, see Rhodes et al., 2004). Second, planning complex trajectories takes longer than simple reaching movements to a certain spatial position (Wong et al., 2015). Finally, relying on on-line planning leads to a reduction in movement latency time (Orban de Xivry et al., 2017) and therefore results in shorter pauses between consecutive movements.

## Preliminary Motor Training and Its Impact on Solving the Nine-Dot Problem

We conducted three experiments. The primary goal of Experiment 1 was to assess the method’s potential explanatory power and, at the same time, to assay the patterns of motor activity related to on-line and off-line modes of motor planning. The second and third experiments were designed to uncover the potential impact of preliminary motor training on the motor output of the successful and unsuccessful problem solvers.

A known way to boost the probability of the correct solving of the nine-dot problem is to ask participants to precede their attempts to solve the problem by motor training – by drawing those line segments that are part of the correct solution (Weisberg and Alba, 1981, Experiment 2; Lung and Dominowski, 1985; Chronicle et al., 2001, Experiment 3). Using preliminary motor training allows us to uncover the movements (and combinations of thereof) that play an important role in problem-solving and to shed light on both the nature and the sources of the difficulties the problem solvers met (Kershaw and Ohlsson, 2004). In particular, we believe that using motor training also allows for studying the contribution made by the two modes of motor planning mentioned above.

The traditional variant of preliminary motor training does not distinguish between the instrumental and functional role of motor activity. For example, Kershaw and Ohlsson (2004, Experiment 1) varied two factors that were related to preliminary motor training. These factors were (i) the presence/absence of non-dot turns, i.e., actual abrupt changes in movement direction taking place outside the nine dots area and (ii) the presence/absence of perceptual cues for non-dot turns. In order to accomplish the task, a solver has to arrange the required movements while keeping in mind the verbal instructions (“connect the dots by straight lines”). This mode of motor training involves both kinds of motor planning (on-line and off-line) as well as instrumental aspects of a motor activity.

In order to discriminate between the instrumental and functional roles that preliminary motor training might play, we studied the impact of the training on the solving process in each of the following two conditions: in the “no task” condition (movements played a predominantly instrumental role) and in the context of a task in which movements played both an instrumental and functional role. In our Experiment 2, we used traditional preliminary motor training in which participants practiced drawing pairs of consecutive segments with their connection point (vertex) situated out of the nine-dot display [usually referred to as “non-dot turns” (Kershaw and Ohlsson, 2004)]. These line drawings are known to be the crucial elements of the correct solution for the nine-dot problem. This kind of training involved both off-line and on-line planning modes. In Experiment 2, participants were asked to connect dots by two connected straight-line segments. These line segments were oriented at an angle that could take two different values. Here the preliminary motor training was explicit and took place in the context of a task that was relevant to the upcoming problem. In our Experiment 3, the preliminary motor training was implicit and proceeded in the context of a task that was seemingly irrelevant to the nine-dot problem. In this Experiment, we used a modified version of the implicit learning paradigm, in which participants remained unaware of either the results of the learning or the learning itself (Nissen and Bullemer, 1987; Cleeremans et al., 1998). Applying this experimental technique makes it possible to estimate the effect of specific movements on how efficient solvers are in finding the solution to the problem. In Experiments 2 and 3, we tested hypotheses on how preliminary motor training, which presumably played a functional role in Experiment 2 and an instrumental role in Experiment 3, affects the motor activity of a problem solver and hence the effectivity of solving the problem. In sum, the goal of the present study was to identify movement sequences executed during attempts to solve the nine-dot problem. To this end, the experimental procedure was modified so that it allowed for the recording of the motor activity with a tablet computer and for the extraction of informative parameters of this activity such as the times taken for drawing line segments and the duration of pauses between successive movements.

## Experiment 1

In the first study, we attempted to identify the differences between successful and unsuccessful nine-dot problem solvers by using several variables that characterized the motor activity of solvers.

### Methods

#### Participants

Forty-five volunteers (35 women; 18–21 years old, *M* = 19.32; *SD* = 0.59) from Moscow universities (RANEPA, NRU HSE) participated in the experiment in return for course credits. Six participants were excluded from the further analysis because in the post-experimental survey they reported that the nine-dot problem was familiar to them. Three participants solved the nine-dot problem unconventionally (angles were not equal to 45 degrees) and were excluded from the analysis too.

This study was carried out in accordance with the recommendations of institutional guidelines of the ethics committee of the Department of Psychology of RANEPA (Russian Academy of National Economy and Public Administration). The protocol was approved by the ethics committee of the Department of Psychology of RANEPA. All participants gave written informed consent in accordance with the Declaration of Helsinki.

#### Apparatus and Stimuli

Conducting experiments, we used a custom program in Delphi language on an Asus tablet (10.1-inches screen diagonal; 1280 pixels × 800 pixels, PPI = 143; Intel Atom X5-Z8500 quad-core processor clocked at 1.44 GHz; operating system Windows 10). The software presented the nine-dot problem and recorded the motor activity of participants trying to solve the problem. The participants used the tip of their index finger to draw line segments on the screen of the tablet. All movements left visible traces on the tablet screen.

At the beginning of the experiment, the program recorded the age, sex, and participant identification number. Then it presented the instructions and an image of nine dots. Nine black dots were presented in the form of a “square” in the center of a tablet’s screen. Each dot was 10 mm in diameter. The distance between neighboring dots was 15 mm vertically and horizontally.

#### Design and Procedure

Participants solved the nine-dot problem while sitting at a table. The tablet was on the table in front of them. Participants were asked to solve the nine-dot problem. First, they were presented with on-screen instructions (in Russian): “Please connect all 9 dots by drawing four straight lines with the tip of your index finger without taking your index finger off the screen of the tablet.”

No standard home position for the index finger was used so participants were free to start from any point on the screen. As soon as participants began drawing lines, the program collected raw data of their motor activity (coordinates of all points in drawn lines in pixels and the processor time corresponding to each coordinate value in milliseconds). In the upper left corner of the screen were two buttons: “Save” and “Next trial.” If participants succeeded in solving the problem, they pressed «Save». However, if they failed to solve the problem, they pressed “Next trial” and tried to solve it again. The experiment was limited to 100 trials, and if a participant did not solve the nine-dot problem within this number of trials, he or she was considered unsuccessful. In addition to the parameters of motor activity, the solution time, solution rate and a number of used trials were also recorded. The experiment was carried out individually. At the end of the experiment, participants were asked whether they were familiar with the nine-dot problem. If they responded positively, they were excluded from further analysis.

The duration of pauses between lines in milliseconds and the duration of one line drawing in milliseconds were the dependent variables. The grouping variables were the solution rate and the stage of the nine-dot problem-solving. The stages of problem-solving were set by dividing the total number of trials of each participant into three equal parts (first, second, and third). A similar way of analyzing data was used in studies of oculomotor activity during the insight solution (Knoblich et al., 2001).

#### Data Analysis

We used Octave/Matlab custom software to analyze movement recordings. The analysis proceeded through several successive stages (Korneev and Kurgansky, 2013). In the first stage, we used the linear interpolation technique to convert the original time series into the time series *x*(*n*) and *y*(*n*) equally spaced in time (here *n* stands for a discrete time). In the second stage, the *x*(*n*) and *y*(*n*) series were smoothed with the 2nd order Butterworth low-pass filter with cut-off frequency of 5 Hz. A forward and reverse filtering was applied to the time series to preserve the original phase spectrum. The resultant smooth planar trajectory {*x*(*n*), *y*(*n*)} was used to compute instantaneous tangential velocity *v*(*n*). In the third stage, the entire movement recording was broken into a sequence of successive submovements. To that end, all the local peaks in *v*(*n*) time series were found. In order to reduce the noise caused by physiological tremor and small corrective submovements, any peak whose height was less than 10% of the height of the tallest peak was excluded from further analysis. For each of the valid tangential velocity peaks its margins were determined. It was assumed that *v*(*n*) is a monotonically increasing function of the discrete time n on the left-hand side of a peak corresponding to a submovement while it is a monotonically decreasing function of n on the right slope of the peak. Therefore, the leftmost time point of increasing slope and the rightmost time point of the decreasing slope were taken as the beginning and the end of the peak. As a result of the above procedure, all movement recordings were broken into a sequence of peaks (corresponding to non-overlapping fractions of submovements).

In the final stage, all extracted submovements were assigned to a certain line segment. For each extracted peak, a vector pointing from the starting to the end position was computed. Any pair of adjacent vectors were considered as belonging to the same line segment if the angle between these two vectors did not exceed a chosen critical angle (usually 30 degrees). Potentially, the sequence of extracted peaks and their assignment to a particular trajectory segment can be used in order to compute a number of variables that constitute very detailed multidimensional characteristics of a motor activity of a problem solver. In the present work, we used two variables which are referred to throughout the paper as “movement time” and the “pause duration.” The movement time variable corresponds to the mean time across all segments required to draw a single line segment. This value does not include the time of staying motionless (or moving very slowly with a velocity below some predefined threshold) in the joints of the trajectory. The latter time is characterized by the second variable, pause duration. This variable is computed by averaging all the particular pauses detected during drawing a sequence of line segments. The reason why we limited our scope to these two variables is that they are presumably related to the on-line and off-line motor planning modes, correspondingly.

### Results

#### Movement Time

The first question is whether solvers and non-solvers differed in the movement time during line drawing at different stages (first, second, and third) of the solution. The overall solution rate was 52.8% (19 solvers and 17 non-solvers). A 2 × 3 repeated measures ANOVA with SUCCESS (solvers and non-solvers) as a between-subjects factor and STAGE (first, second, and third) as a within-subjects factor revealed no significant main effects (*p* = 0.09 and *p* = 0.27, respectively). However, there was a significant interaction between factors of SUCCESS and STAGE [*F*(2,68) = 3.3, *p* = 0.044, $\eta_{p}^{2}$ = 0.09]. Figure 2 shows mean movement time for solvers and non-solvers in the three stages of the nine-dot problem solving.

A series of *t*-tests for independent samples were conducted to clarify at which stages solvers and non-solvers differ (Table 1). There were no differences between solvers and non-solvers at the first (*p* = 0.63) and the second stages (*p* = 0.38). But we found that solvers drew lines significantly more slowly than non-solvers at the third stage [*t*(21) = 2.39, *p* = 0.03, *d* = 0.78]. We used Welch’s *t*-test because variances were unequal.

**Table 1:** Mean and standard deviation of movement time in the three stages of the nine-dot problem solving.

	Solvers			Non-solvers			
			
	*M*	*SD*	*M*_total_	*M*	*SD*	*M*_total_	*M*_stages_
First stage	1142.3	642.16		1059.73	287.89		1103.31
Second stage	1108.06	625.32	1252.11	955.13	337.95	974.58	1035.84
Third stage	1505.96	1052.35		908.87	271.79		1223.99


#### Pause Duration

The next question is whether solvers and non-solvers differed in the pause duration between line drawing at different stages (first, second, and third) of the solution (Table 2). A 2 × 3 repeated measures ANOVA with SUCCESS (solvers and non-solvers) as a between-subjects factor and STAGE (first, second, and third) as a within-subjects factor revealed no significant main effect of SUCCESS (*p* = 0.55), STAGE (*p* = 0.18) or interaction of SUCCESS and STAGE (*p* = 0.13).

**Table 2:** Mean and standard deviation of pause duration in the three stages of the nine-dot problem solving.

	Solvers			Non-solvers			
			
	*M*	*SD*	*M*_total_	*M*	*SD*	*M*_total_	*M*_stages_
First stage	749.6	450.38		1114.04	654.85		931.82
Second stage	1118.07	944.16	924.93	1089.08	912.91	1065.43	1103.58
Third stage	907.12	896.49		993.16	611.79		950.14


### Discussion

These results show that the way off-line planning mode was used did not change across successive stages of the process of solving the nine-dot problem either in successful or in unsuccessful problem solvers. This conclusion is supported by the absence of significant changes in pause duration across successive stages of the solving process. However, we observed a significant difference between successful and unsuccessful problem solvers in the movement time parameter at the final third stage of the solving process (supported by the presence of a statistically significant SUCCESS and STAGE interaction). This finding suggests that successful solvers relied more on on-line planning than their unsuccessful peers. The results of this experiment show that analyzing actual movement patterns is capable of providing new information on the processes underlying the solving of insight problems.

Similar differences between successful and unsuccessful problem solvers were found using eye tracking during the final stage of the problem solving (Knoblich et al., 2001). They found that it was the third stage of the solving process in which the average duration of long fixations spent on crucial elements in matchstick arithmetic problems was significantly longer in successful than in unsuccessful problem solvers. The explanation suggested by Knoblich et al. (2001) involved a re-structuring the inner representation of the problem, which in turn caused a re-distribution of attention from irrelevant to relevant task conditions. Thus, one may say that they considered the motor activity of problem solvers from the instrumental perspective, i.e., as something caused by the functioning of mental mechanisms. However, our data showed that successful nine-dot problem solvers mostly rely on on-line planning, thus pointing to the functional role of motor activity. The experiments that follow are designed to study the functional role of motor activity.

## Experiment 2

Experiment 1 suggests a link between the success in solving the nine-dot problem and the on-line mode of motor planning. Experiment 2 is aimed at verifying whether the on-line planning can causally influence successfulness of the nine-dot problem solving. In order to elucidate the role of motor activity in the successful solving of the nine-dot problem we used a well-known method – preliminary motor training, i.e., practicing isolated constituents of a correct solution of a problem. If such preliminary training has a positive impact on finding the problem solution (Weisberg and Alba, 1981; Lung and Dominowski, 1985) then this method allows studying not only the instrumental role of motor activity but also its functional role. We expected that the functional role of motor activity would be most noticeable in the case of practicing non-dot turn, which is one of the key elements of the correct nine-dot problem solution. A non-dot turn is a turn made by the pen tip outside the square area that contains all nine dots. This element of the solution was considered by Kershaw and Ohlsson (2004). The purpose of the second experiment was to study how two factors that characterize the preceding motor activity, practicing dot vs. non-dot turns and practicing turns with the solution-relevant (45 degrees) vs. solution-irrelevant (26.6 degrees) angles, influence solving of the nine-dot problem.

### Methods

#### Participants

A total of 74 volunteers (65 women; 17–28 years old, *M* = 19.0; *SD* = 0.59) from Moscow universities (RANEPA, RSUH) participated in the experiment in return for course credits. Five participants were excluded from the further analysis because their solution to the nine-dot problem, although correct, was geometrically unconventional (angles were not equal to 45 degrees). Five participants were removed from the further analysis because they solved the nine-dot problem in less than three trials. One participant who had mean values of pauses duration more than 3 standard deviations from the average was excluded too.

This study was carried out in accordance with the recommendations of institutional guidelines of the ethics committee of the Department of Psychology of RANEPA. The protocol was approved by the ethics committee of the Department of Psychology of RANEPA. All participants gave written informed consent in accordance with the Declaration of Helsinki.

#### Apparatus and Stimuli

The nine-dot problem was administered the same way as in Experiment 1 with the only exception of the tablet computer model. In this experiment, we used an HP tablet (10.1-inches screen diagonal; 1280 pixels × 800 pixels, PPI = 143; the Intel Atom Z3735G quad-core processor clocked at 1.33 GHz; operating system Windows 10).

**FIGURE 3:**
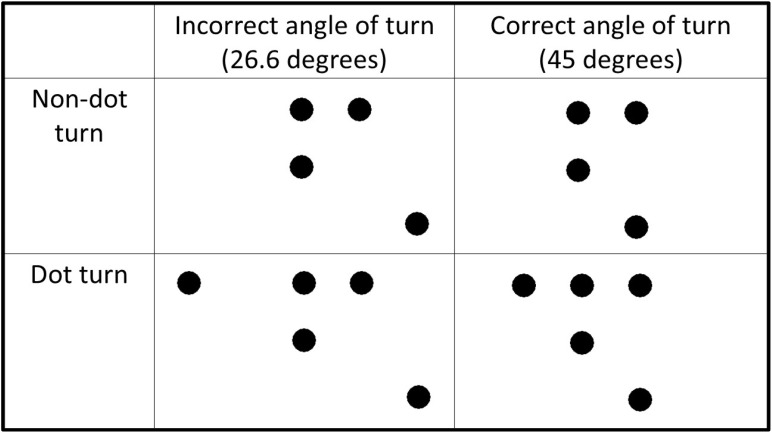
Types of motor training tasks. Motor training tasks were divided into four groups: non-dot turn and incorrect angle of turn (Group 1); non-dot turn and correct angle of turn (Group 2); dot-turn and incorrect angle of turn (Group 3); and dot-turn and correct angle of turn (Group 4).

Before solving the nine-dot problem, participants solved a series of motor training tasks. These were presented on the tablet using the same software as in Experiment 1. Participants were presented with four or five dots, which were arranged so that two straight lines with a turn of 45 or 26.6 degrees could connect them (see Figure 3). Each motor training task was repeated 4 times with the angle vertex pointing to different directions (angle up, angle down, angle to the right, and angle to the left).

#### Design and Procedure

Participants were asked to solve several motor training tasks. In the first four tasks, it was necessary to connect dots with two lines, without lifting the index finger from the screen of the tablet. In the upper left corner of the screen, there were two buttons: “Done” and “Next trial.” If participants succeeded in solving a task, they pressed «Done». However, if they failed to solve a task, they pressed “Next trial” and tried to solve it again. The number of trials for these motor training tasks was unlimited.

Participants were randomly distributed into four groups. In Group 1, motor training tasks required participants to perform a non-dot-turn with 26.6 degrees (see Figure 3). In Group 2, motor training tasks required participants to perform a non-dot-turn with 45 degrees. In Group 3, motor training tasks required participants to perform a dot-turn with 26.6 degrees. And in Group 4, motor training tasks required participants to perform a dot-turn with 45 degrees. Within the groups, the sequence of presentation of tasks was random. After these tasks, participants proceeded to the nine-dot problem. The procedure for solving the nine-dot problem was the same as in Experiment 1. At the end of the experiment, participants were asked whether they used their experience in solving the first four tasks during the nine-dot problem-solving and whether they were familiar with the nine-dot problem.

### Results

To control whether the non-dot-turn training and correct angle of turn training affected problem solving performance, we compared two groups of participants in terms of solution rate.

#### Impact of the Preliminary Motor Training on the Performance: Non-dot Turn vs. Dot Turn

The overall solution rate of the nine-dot problem was 57.8%; 37.5% in the dot-turn training group and 78.1% in the non-dot-turn training group (see Table 3). According to Chi-square test, the association between training type (non-dot turn vs. dot turn) and solution rate was statistically significant [χ^2^(1, *N* = 64) = 10.83, *p* = 0.001].

**Table 3:** Solution rate in two experimental groups with and without non-dot-turn at the motor training.

	Non-solvers	Solvers	Total
Dot-turn training	20	12	32
Non-dot-turn training	7	25	32
Total	27	37	64


#### Impact of the Preliminary Motor Training on the Performance: Correct vs. Incorrect Angle

There were 51.6% of successful solutions to the nine-dot problem in the incorrect angle (26.6 degrees) training group and 63.6% in the correct angle (45 degrees) training group (Table 4). According to Chi-square test, the association between training type (correct vs. incorrect angle of turn) and solution rate was not significant: χ^2^(1, *N* = 64) = 0.95, *p* = 0.33.

**Table 4:** Solution rate in two experimental groups with and without the correct angle of turn at the motor training.

	Non-solvers	Solvers	Total
Correct angle training	12	21	33
Incorrect angle training	15	16	31
Total	27	37	64


#### Movement Time

**FIGURE 4:**
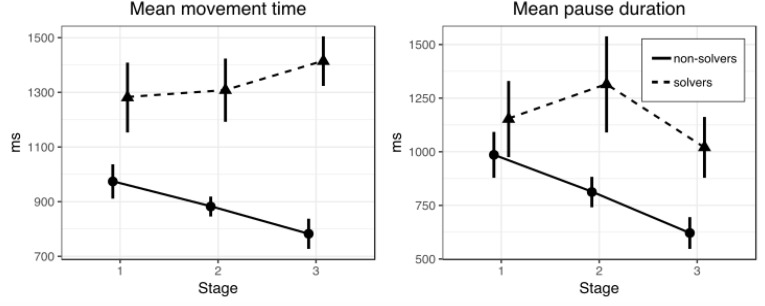
Mean movement **(left)** and pause **(right)** time in three stages of the nine-dot problem solving (Experiment 2). Bars represent within-subject 95% confidence intervals.

As in Experiment 1, we tried to find similar differences between solvers and non-solvers in the movement time during line drawing at different stages (first, second, and third) of the solution. Movement times were subjected to a 2 × 3 repeated measures ANOVA with SUCCESS (solvers and non-solvers) as a between-subjects factor and STAGE (first, second, and third) as a within-subjects factor. This analysis revealed a significant main effect of SUCCESS [*F*(1,61) = 18.38, *p* = 0.001, $\eta_{p}^{2}$ = 0.23] and a significant interaction between factors of SUCCESS and STAGE [*F*(2,122) = 6.23, *p* = 0.003, $\eta_{p}^{2}$ = 0.09]. There was no significant main effect of STAGE (*p* = 0.96). Figure 4 shows mean movement time for solvers and non-solvers in all three stages of the nine-dot problem solving after motor training.

A series of *t*-tests for independent samples were conducted to clarify at which stages solvers and non-solvers differ (Table 5). We used Welch’s *t*-test as variances are unequal. There were no differences between solvers and non-solvers at the first stages (*p* = 0.08). But we found that at the second [*t*(54) = 3.39, *p* = 0.03, *d* = 0.98] and at the third [*t*(50) = 6.07, *p* < 0.001, *d* = 1.49] stages solvers drew lines significantly more slowly than non-solvers.

**Table 5:** Mean and standard deviation of movement time in the three stages of the nine-dot problem solving after motor training.

	Solvers			Non-solvers			
			
	*M*	*SD*	*M*_total_	*M*	*SD*	*M*_total_	*M*_stages_
First stage	1288.13	656.49		1035.28	323.82		1180.36
Second stage	1346.97	572.98	1376.08	927.93	302.30	928.39	1168.36
Third stage	1493.15	578.97		821.97	261.85		1207.07


#### Impact of Motor Training on Movement Time

Movement time was subjected to a 2 × 2 ANOVA with NON-DOT TURN (non-dot turn training and dot turn training) and ANGLE (correct angle training and incorrect angle training) as a between-subjects factors. This analysis revealed a significant main effect of NON-DOT TURN [*F*(1,61) = 7.8, *p* = 0.007, $\eta_{p}^{2}$ = 0.12], but no significant main effect of ANGLE (*p* = 0.22) and no interaction of NON-DOT TURN and ANGLE (*p* = 0.76) were found (Table 6).

**Table 6:** Movement time in four experimental groups with different types of motor training.

	Dot-turn training		Non-dot-turn training		Total	
			
	*M*	*SD*	*M*	*SD*	*M*	*SD*
Correct angle training	888.32	279.54	1261.08	410.59	1081.13	395.61
Incorrect angle training	1128.33	323.45	1412.24	859.76	1270.29	655.05
Total	1016.33	322.45	1339.10	673.92		


#### Pause Duration

Also, as in Experiment 1, we tried to find similar differences between solvers and non-solvers in the pause duration between lines drawing at different stages of the solution. A 2 × 3 repeated measures ANOVA with SUCCESS (solvers and non-solvers) as a between-subjects factor and STAGE (first, second, and third) as a within-subjects factors revealed significant main effects of SUCCESS *F*(1,61) = 4.49, *p* = 0.038, $\eta_{p}^{2}$ = 0.07, and STAGE *F*(2,122) = 3.18, *p* = 0.045, $\eta_{p}^{2}$ = 0.05. The interaction between factors of SUCCESS and STAGE was also significant, *F*(2,122) = 4.32, *p* = 0.015, $\eta_{p}^{2}$ = 0.07. Figure 4 shows means for pause duration for solvers and non-solvers in all three stages of the nine-dot problem solving after motor training.

A series of *t*-tests for independent samples were conducted to clarify at which stages solvers and non-solvers differ (Table 7). We used Welch’s *t*-test, as variances are unequal. There were no differences between solvers and non-solvers in the first stages of the solution (*p* = 0.69). But we found that at the second [*t*(46) = 2.88, *p* = 0.01, *d* = 0.5] and at the third [*t*(55) = 2.27, *p* = 0.03, *d* = 0.56] stages solvers make significantly longer pauses between drawing lines than non-solvers.

**Table 7:** Mean and standard deviation of pause duration in the three stages of the nine-dot problem solving after motor training.

	Solvers			Non-solvers			
			
	*M*	*SD*	*M*_total_	*M*	*SD*	*M*_total_	*M*_stages_
First stage	1067.9	642.2		1137.9	702.6		1097.7
Second stage	1390.2	1064.4	1164.6	825.3	395.7	885.4	1149.4
Third stage	1035.6	754.3		693.0	414.6		889.6


#### Impact of the Motor Training on Pause Duration

Pause duration was subjected to a 2 × 2 ANOVA with NON-DOT TURN (non-dot turn training and dot turn training) and ANGLE (correct angle training and incorrect angle training) as a between-subjects factors. This analysis revealed no significant main effect of NON-DOT TURN (*p* = 0.08), ANGLE (*p* = 0.23) and interaction of NON-DOT TURN and ANGLE (*p* = 0.41) (Table 8).

**Table 8:** Pause duration in four experimental groups with different types of motor training.

	Dot-turn training		Non-dot-turn training		Total	
			
	*M*	*SD*	*M*	*SD*	*M*	*SD*
Correct angle training	830.02	478.01	1048.81	455.88	943.18	471.57
Incorrect angle training	1335.57	905.26	1139.95	647.48	1237.76	780.55
Total	1099.64	769.49	1095.85	555.65		


### Discussion

Experiment 2 showed that preliminary motor training involving non-dot turns resulted in more success in finding a correct solution as compared to the training that did not involve these turns. Practicing a task-relevant turn of 45 degrees was no better than practicing a task-irrelevant turn of 26.6 degrees. Although the latter finding is in line with previous studies (Kershaw and Ohlsson, 2004), it does not support our hypothesis of the superiority of a task-relevant angle of 45 degrees. It may well be that the direction of the upcoming movement is an essential part of the motor plan since it helps to transcend the perimeter of the visible nine dot display whereas angles between two successive segments are not parts of the movement plan.

When comparing the results of the present experiment with those of Experiment 1, one can notice that motor training caused the difference between successful and unsuccessful problem solvers in parameters quantifying on-line planning not only at the final stage of the solution but also at the second stage. This finding suggests that being affected by preliminary motor training, successful problem solvers tended to invoke an on-line mode of movement planning at earlier stages of the process of solving the nine-dot problem. Besides, in this experiment, we found a difference between successful and non-successful problem solvers in pause duration during the second and the third stages of the problem-solving process. The latter finding suggests that successful solvers rely to a greater extent on off-line planning than their unsuccessful peers.

Results of this experiment suggest that processes underpinning motor planning make a substantial contribution to the successful solving of the nine-dot problem. We found that successful problem solvers showed greater movement time (associated with on-line planning) as well as greater pause duration (associated with off-line planning) than their unsuccessful counterparts. This finding is in accordance with the view that both kinds of planning contribute to the successful solving of the nine-dot problem.

A slowing down of drawing lines which is found in successful problem solvers suggests that they spend progressively more time preparing the rest of the ongoing and upcoming line segment amidst executing a current movement. It should be noted that the on-line planning mode leads to resource re-distribution favoring the remaining part of the movement being executed. Since the movement’s starting point and line direction are set by already executed movement(s), i.e., by the already completed fraction of the line being drawn, it is the choice of a final position that becomes the focus of the planning process. In its turn, the final position becomes the starting position for the next line segment. Therefore, planning a final position for a current line segment might be accompanied by the planning of a specific angle for the next turn if a direction of the next line is also chosen.

We also observed a progressive growth in pause duration along the solution process for successful nine-dot problem solvers. This observation suggests that apart from on-line planning activity, these solvers also used off-line planning in multiple attempts to arrange sequences of line segments required for the nine-dot problem solution in the mental space. One might think that on-line and off-line modes of planning are mutually exclusive. Our results showed that this is not the case. Instead, solvers seem to rely on both modes of planning, with the heaviest use of both modes being observed at the late stages of the solution process. One might hypothesize then that using on-line mode of planning lays the ground for the successful use of off-line planning. Early inadequate representations of the nine-dot problem are constrained by certain perceptual templates (e.g., arrangement of nine dots inside the square area) which are used for off-line planning of line segments. Using on-line planning allows for relaxing these constraints and, after a while, it allows for lifting them altogether, thus clearing the way for the adequate off-line planning correct solution of the nine-dot problem.

## Experiment 3

In the third experiment, we aimed to test the effectiveness of implicit hints on the solution of the nine-dot problem. Before the nine-dot problem, participants performed a preparatory task, which included exact movements making up one of the possible solutions of the target problem. The preparatory task involved a serial reaction time task which masked target movements with intervening irrelevant movements, making the hint implicit. This task was widely used to study implicit motor learning (Nissen and Bullemer, 1987; Cleeremans et al., 1998). The typical paradigm usually includes several locations presented to a participant. In each trial, participants are asked to press as fast as possible a button corresponding to the location where the target stimulus appeared. If a sequence of target locations follows some complex regularity, participants demonstrate sensitivity to it (i.e., faster responses to regular vs. irregular target locations) but fail to report the regularity or even do not notice that there was some regularity at all. We expected that participants would implicitly learn the sequence, which in turn would lead to a higher probability of successful problem solving since the learned sequence constitutes the correct solution for the nine-dot problem.

### Methods

#### Participants

Fifty-eight volunteers (47 women, 17–20 years old, *M* = 18.0, *SD* = 0.71) took part in the experiment. All of them were RANEPA students and participated for a part of course credit.

This study was carried out in accordance with the recommendations of institutional guidelines of the ethics committee of the Department of Psychology of RANEPA. The protocol was approved by the ethics committee of the Department of Psychology of RANEPA. All participants gave written informed consent in accordance with the Declaration of Helsinki.

#### Apparatus and Stimuli

**FIGURE 5:**
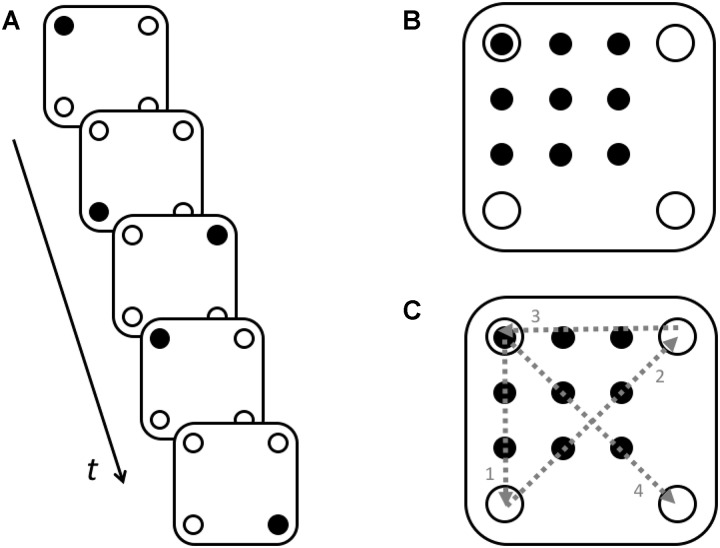
Displays in Experiment 3. **(A)** Displays sequence in the training task (one regular sequence). Black dot was a target dot which must be reached with a finger. **(B)** The spatial arrangement of the training task stimuli and the nine-dot problem. **(C)** The relationship between a series of movements in the regular sequence of the training task and one of the nine-dot problem solutions.

The nine-dot problem was administered the same way as in Experiments 1 and 2. However, it was preceded by an additional task. The setup was presented on a tablet using the same software as the abovementioned experiments. Participants were presented with a series of displays with four dots, three of which were empty and one – black. Participants had to trace a black dot moving their finger on the tablet’s screen from old black dot position to a new black dot position (see Figure 5). The upper left dot was placed in the same position as the upper left dot in nine-dot problem. The other three dots were placed outside of the nine-dot square, but in those positions, which must be crossed in correct nine-dot problem solution.

#### Design and Procedure

Participants were told that they were going to solve several tasks. The first task was to catch the black dot among white dots with an index finger of the dominant arm. When the task was launched, participants were presented with the first display and had to start the task. When they touched the black dot, a new display appeared with the new position of the black dot. Participants were instructed to move the finger toward the new dot without lifting the finger from the screen.

Unbeknownst to participants, this task consisted of 60 series of 5 displays in each. Thirty series were regular (repeating the same sequence of black dot positions) and thirty series were random (five displays presented the random position of black dot). Random and regular series followed one by one. The first series was random, then regular, then random and so on. The sequence of displays was programmed such that black dot did not appear in the same dot place twice in a row. The random series contained the same number of every position for the black dot as the regular series, for example if the regular series was 1-3-2-1-4, in the random series, black dot had to appear once in the first, third and fourth positions and twice in the first position. Thus, this task may be seen as a variant of a serial reaction time task (Nissen and Bullemer, 1987).

Participants were randomly distributed in two groups. In the first group (*N* = 29), regular series required participants to perform exactly the same movements that are needed for one of the successful solutions of nine-dot problem, thus we will refer to this group as “Relevant training” group (see Figure 5). The second group (*N* = 29) was divided into two subgroups (*N* = 15 and 14) with different regular series. In both subgroups, regular series contained another combination of movement which were useless in nine-dot problem solution, thus “Irrelevant training” group.

After that task, participants proceeded to the nine-dot problem. The procedure of the nine-dot problem solving was the same as in Experiment 1. In the end of the experiment, participants were asked whether they noticed any regularities in the first task. If they responded positively, they were asked to explain what sequence of dots they noticed.

### Results

#### Learning

To evaluate learning, we deleted 1.5% of fastest and 1.5% of slowest responses for every participant. All the trials were averaged by blocks of 10 trials (2 series: random + regular) for every participant. The first block was deleted from the analysis as participants were very slow on the first trials. The first five trials were always random. Learning was assessed by fitting a linear regression with the number of blocks as predictor and RT as the dependent variable. The quadratic model indicated a better fit than a linear one (*F* = 28.43, *p* < 0.001), indicating the non-linear decrease of RTs with practice. The learning of regular sequence was examined by paired *t*-test (regular vs. random sequences), *t*(57) = 7.15, *p* < 0.001, indicating faster movements for regular sequences (*M* = 566 ms, *SD* = 63) in comparison to random sequences (*M* = 584 ms, *SD* = 66). None of the participants correctly reported the sequence of regular displays when asked.

#### The Effect of Training

The number of successful solutions in the Relevant training group was 7 (24.1%), and in the Irrelevant training group it was 14 (48.3%) (Table 9). The difference in the proportion of successful solutions in two groups did not reach significance according to Chi-squared test with Yates continuity correction, χ^2^(1) = 2.69, *p* = 0.101. To assess a non-specific effect of training, we compared solution rates in each group with the solution rates from Experiment 1 (52.8% successful solutions). The Relevant training group had significantly lower proportion of successful solutions [χ^2^(1) = 9.56, *p* = 0.002], whereas the Irrelevant training group did not differ from the group of participants in Experiment 1 [χ^2^(1) = 0.24, *p* = 0.626].

**Table 9:** Solution rate in two experimental groups with relevant and irrelevant training.

	Non-solvers	Solvers	Total
Relevant training	22	7	29
Irrelevant training	15	14	29
Total	37	21	58


#### Movement Time

**FIGURE 6:**
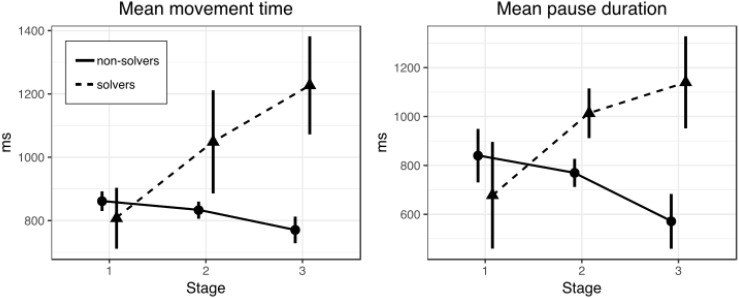
Mean movement **(left)** and pause **(right)** time in three stages of the nine-dot problem solution (Experiment 3). Bars represent within-subject 95% confidence intervals.

As in previous experiments, we analyzed movement patterns in the nine-dot problem solution. First, we analyzed the difference in movement times between solvers and non-solvers. A 2 × 3 repeated measures ANOVA with SUCCESS (solvers and non-solvers) as a between-subjects factor and STAGE (first, second, and third) as a within-subjects factor revealed significant main effects of SUCCESS, *F*(1,56) = 4.85, *p* = 0.032, $\eta_{p}^{2}$ = 0.08, and STAGE, *F*(2,112) = 6.60, *p* = 0.002, $\eta_{p}^{2}$ = 0.11. The interaction between SUCCESS and STAGE was also significant, *F*(2,112) = 15.48, *p* < 0.001, $\eta_{p}^{2}$ = 0.22, indicating different dynamics in movement time in solvers and non-solvers across three stages. Pairwise comparisons using *t*-test revealed that there was no difference between solvers and non-solvers at the first (*p* = 0.517) stage. The difference was marginally significant at the second stage (*p* = 0.056) and significant at the third stage (*p* = 0.006), indicating that solvers gradually became slower than non-solvers (Table 10). Figure 6 shows mean movement times for successful and unsuccessful solvers in all three stages of the nine-dot problem solving.

**Table 10:** Mean and standard deviation of movement time in the three stages of the nine-dot problem solving.

	Solvers			Non-solvers			
			
	*M*	*SD*	*M*_total_	*M*	*SD*	*M*_total_	*M*_stages_
First stage	807.32	371.7		861.38	257.74		841.81
Second stage	1048.59	537.22	1027.68	833.08	304.57	821.67	911.11
Third stage	1227.12	654.07		770.55	298.06		935.86


Three-way GROUP × SUCCESS × STAGE interaction was not significant (*p* = 0.68), indicating the similar pattern of results between two groups (see Tables 11, 12). Two-way GROUP × SUCCESS and GROUP × STAGE interactions were also non-significant (*p* = 0.15 and *p* = 0.56, respectively).

**Table 11:** Mean and standard deviation of movement time in the three stages of the nine-dot problem solving after relevant training.

	Solvers			Non-solvers			
			
	*M*	*SD*	*M*_total_	*M*	*SD*	*M*_total_	*M*_stages_
First stage	648.93	216.11		877.16	282.1		822.01
Second stage	794.85	167.37	843.37	828.52	361.35	823.62	820.39
Third stage	1086.34	525.03		765.19	318.9		842.71


**Table 12:** Mean and standard deviation of movement time in the three stages of the nine-dot problem solving after irrelevant training.

	Solvers			Non-solvers			
			
	*M*	*SD*	*M*_total_	*M*	*SD*	*M*_total_	*M*_stages_
First stage	886.52	413.21		838.23	224.67		561.54
Second stage	1175.47	615.69	946.79	837.76	206.36	991.17	1001.83
Third stage	778.39	275.15		1297.51	717.59		1029


#### Pause Duration

The same model was run for pause duration between lines drawing. A 2 × 3 repeated measures ANOVA with SUCCESS (solvers and non-solvers) as between-subjects factor and STAGE (first, second, and third) as within-subjects factor revealed no significant main effects. The two-way interaction was significant, *F*(2,112) = 12.14, *p* < 0.001, $\eta_{p}^{2}$ = 0.18, indicating different dynamics in pause durations in solvers and non-solvers across three stages. By using a *t*-test for pairwise comparisons, we observed no significant difference between solvers and non-solvers at the first (*p* = 0.345) and second stages (*p* = 0.165). But we found that at the third stage solvers made significantly longer pauses than non-solvers, (*p* = 0.008) (Table 13). Figure 6 shows means and corresponding confidence intervals of the pauses time for solvers and non-solvers in three stages of the nine-dot problem solution.

**Table 13:** Mean and standard deviation of pause duration in the three stages of the nine-dot problem solving.

	Solvers			Non-solvers			
			
	*M*	*SD*	*M*_total_	*M*	*SD*	*M*_total_	*M*_stages_
First stage	678.01	398.82		839.94	717.77		781.31
Second stage	1013.28	682.07	943.72	769.43	606.88	726.9	857.72
Third stage	1139.79	851.31		571.33	368.81		777.15


Then, we added GROUP factor (Relevant and Irrelevant training groups) to the model. The three-way interaction between “GROUP × SUCCESS × STAGE” was not significant (*p* = 0.956), thus indicating a similar pattern of results between two groups (see Tables 14, 15). Two-way “GROUP × SUCCESS” and “GROUP × STAGE” interactions were also not significant (*p* = 0.935 and *p* = 0.129, respectively).

**Table 14:** Mean and standard deviation of pause duration in the three stages of the nine-dot problem solving after relevant training.

	Solvers			Non-solvers			
			
	*M*	*SD*	*M*_total_	*M*	*SD*	*M*_total_	*M*_stages_
First stage	645.58	402.28		852.38	863.21		802.46
Second stage	836.66	646.56	822.34	662.2	606.62	663.21	704.31
Third stage	984.77	758.8		475.06	343.22		598.09


**Table 15:** Mean and standard deviation of pause duration in the three stages of the nine-dot problem solving after irrelevant training.

	Solvers			Non-solvers			
			
	*M*	*SD*	*M*_total_	*M*	*SD*	*M*_total_	*M*_stages_
First stage	694.23	411.3		821.68	454.39		760.15
Second stage	1101.58	705.39	1004.37	926.07	592.04	820.09	1011.13
Third stage	1217.3	910.95		712.51	370.34		


### Discussion

In Experiment 3, we aimed to test, whether non-specific movement training would result in a change of the nine-dot problem solution. During training, participants performed regular sequential movements more quickly than irregular, which means that movements series was learned by them. In the Relevant training group, the regular sequence was identical to one of the solutions of the nine-dot problem, and as such, we expected that participants in this group would be more successful in the nine-dot problem. However, this was not the case as the Irrelevant training group participants solved the task successfully more often than Relevant training participants. Further statistical analysis showed, however, that this difference was not significant. In comparison to the Experiment 1, which had identical nine-dot problem session, the Irrelevant training group showed no significant difference in solution rates, whereas the Relevant training group had the significantly lower proportion of successful solutions than in Experiment 1. We don’t think this result can be explained by the non-specific effect of training. A more probable interpretation is related to the overall lower solution rate in both groups in Experiment 3 than in Experiment 1. We then analyzed movement time and pause time depending on the solution success and group. In both cases, we observed the interaction between solution success and solution stage. Solvers tended to increase both movement times and pause times whereas non-solvers tended to decrease both movement and pause times. Training type (relevant to the nine-dot problem solution or not) did not affect movement and pauses times.

The latter result (i.e., the finding that preliminary motor training involving an irrelevant task does not influence motor activity during nine-dot problem solving) suggests that no transfer of the correct sequence of line segments acquired during the implicit learning session occurred during the solving of the nine-dot problem. The fact that participants did learn the correct sequence of movements while performing some irrelevant task is in accordance with the view that this sequence of movements played a purely instrumental role while approaching the target problem. However, the merely instrumental role played by motor activities was insufficient to target problem solving since no transfer of the learned sequence to the nine-dot problem was found. Therefore, we can argue that for the successful resolution of the nine-dot problem, the motor activity should also play a functional role.

## General Discussion

### An Overview of Major Findings

Based on a preliminary theoretical analysis, we assumed that investigating on-line vs. off-line motor planning separately might be helpful in explaining the difference between successful and unsuccessful solvers of the nine-dot problem. We computed two quantities which are sensitive to the difference between on-line and off-line planning, the movement time and the pause duration, and then used them in order to compare successful and unsuccessful solvers of the nine-dot problem.

We reported three experiments in this study: Experiment 1 through Experiment 3, all of which showed similar results. All three showed that at the third stage of the solution process (the final one third of the block of trials) the successful solvers showed longer movement time than their unsuccessful counterparts. In Experiment 2, test takers also undertook a preliminary motor training prior to the test session. In this case, successful problem solvers slowed down their movements not only during the final third stage but also during the intermediate second stage. Also, our results indicate that successful problem solvers showed longer between-movement pauses at the final third stage in both Experiments 2 and 3 and at the intermediate second stage in Experiment 2. This result is in accordance with the critical role of the mental lookahead in finding the nine-dot problem solution, a theoretical position formulated by MacGregor et al. (2001). In agreement with the aforementioned study, our results show the increasing involvement of off-line planning (which is similar to the mental lookahead) at the late stages of the nine-dot problem solving.

Results of Experiment 2 do not support our assumption on the greater positive effect of practicing a non-dot turn with the relevant to the problem solution angle of 45 degrees over non-dot turn with an irrelevant angle of 26.6 degrees. Practicing non-dot turns of arbitrary angle actually caused some increase in the rate of successful solutions of the nine-dot problem. This result is in line with the empirical evidence showing an important role that non-dot turns play in successful solution of the nine-dot problem (Kershaw and Ohlsson, 2004; Öllinger et al., 2014). Results of Experiment 3 did not confirm our assumption. We expected that preliminary learning a motor pattern corresponding to a fraction of the nine-dot problem solution would help in solving this problem. However, the results of Experiment 3 suggest that learning a correct sequence of movements in the context of an irrelevant task does not affect a process of the nine-dot problem solving.

### The Impact of Preliminary Motor Training on the Solution of the Nine-Dot Problem

It has been shown that preliminary motor training involving practicing different fractions of the correct solution of the nine-dot problem results in growing effectiveness of solving that problem (Weisberg and Alba, 1981; Lung and Dominowski, 1985). Kershaw and Ohlsson (2004) have come to a similar conclusion specifically regarding non-dot turns. We exploited two kinds of the preliminary motor training, a traditional one, which involved both instrumental and functional role of motor activity (problem solvers were practicing non-dot turns of 45 and 26.6 degrees), and another “implicit” training (participants implicitly learned a sequence of movements corresponding to a correct solution of the nine-dot problem) that took place during multiple attempts to perform an irrelevant task with hidden relevance to the target nine-dot problem. In the latter case, it turned out that the motor activity played an exclusively instrumental role in solving of the target problem.

The results obtained in the present study suggest that a preliminary training causes an increase in effectiveness of the nine-dot problem solving only if the movements involved in this training play a functional role in the solving of the nine-dot problem. It turned out that practicing non-dot turns regardless of their angle boosted the effectiveness of the solving process while the preliminary training, in which motor activity played an instrumental role only, did not affect the percentage of the correct solution of the nine-dot problem.

### The Role of On-Line and Off-Line Planning in the Process of the Nine-Dot Problem Solving

A difference between successful and unsuccessful problem solvers allows for understanding what helps the successful solvers to solve the nine-dot problem. The obtained results from the abovementioned experiments provide valuable information for the analysis of the specific role of the on-line and off-line movement planning modes in the process of solving of that problem as well as their relative contribution to the successful problem solution.

There are two decisions that are to be made during the nine-dot problem solving: a problem solver has to select initial and final finger positions. However, this may be done in two modes. A problem solver might arrange a plan for upcoming motor activity (hand drawing the line segments connecting the dots) by arranging a certain sequence of line segments. These arrangements, i.e., off-line planning, occur in the mental space. The off-line planning has a “long horizon,” meaning that several steps are being planned (MacGregor et al., 2001; Chronicle et al., 2004). However, this process goes in the well-established perceptual framework and does not transcend it. This way of movement planning does not help to go beyond the nine dots area because problem solvers usually select one of the visible dots as the movement final position. The second mode of motor planning is that the planning and execution processes go in parallel, which slows down the overt line drawing. In this case, a problem solver first chooses an initial position and then selects a direction of upcoming motion while the selection of a final position is temporarily postponed. During this process of slow line drawing a problem solver considers a wide range of possible final positions including those outside the visible nine dots area. This mode of motor planning has a wide but short horizon.

The two modes of motor planning, off-line and on-line modes, are not mutually exclusive. At the later stages of the solution process, an intensity of involvement of both planning modes is greater in successful than in unsuccessful problem solvers. Thus, one may infer that both modes of motor planning are required in order to successfully solve the nine-dot problem, each mode playing its specific role. One may hypothesize that the involvement of on-line planning mode gradually modifies the way by which the off-line planning mode operates. At the early stages of the solving process, the off-line planning is constrained by the initial perceptual description of the problem, i.e., its early representation. For example, relying exclusively on the spatial positions of nine dots and their specific arrangement in the form of square leads to all the planned movements start and end positions coincide with the visible dots and reside within the square area. Relying on on-line planning helps to gradually overcome these perceptual constraints, which in turn opens a way for adequate off-line planning and as a result of a successful solution of the nine-dot problem. All the above considerations lead to a conclusion that motor activity in its functional role is crucial for solving the nine-dot problem.

In order to account for the experimental results reported in the present work, we considered the role of two modes of the motor planning, the off-line and on-line modes. We believe that this approach can be generalized to those insight problems whose solutions substantially rely on some form of motor activity (the examples of problems of that sort were mentioned above). Substantial similarities can be found in all problems of that kind. At the early stages of the problem-solving process, an inadequate initial representation of the problem leads to activation of irrelevant motor programs which effectively hinder from finding the problem solution. As an example, an inadequate initial representation of the six matches problem leads to that solvers attempt to solve the problem (i.e., to arrange four equal triangles using six matches) by keeping all possible rearrangements of the matches confined to a single plane (Scheerer, 1963). A correct solution requires arranging matches into a tetrahedron in the three-dimensional space. Initial attempts to solve yet another insight problem, the 8-coin problem, are limited by moving coins along the plane whereas the correct solution requires leaving the plane for the three-dimensional space (Ormerod et al., 2002). Relying on the on-line mode of the motor planning while solving the above-mentioned problems, like in the case of the nine-dot problem, could help to overcome the inadequate initial representations of these problems and allow the solvers to operate in the three-dimensional space. Of course, this possibility requires an experimental verification (see section Future Directions).

The results obtained in this work cannot be easily accounted for by dominant theories of insight problem solving. The representational change theory is based on the chunk decomposition, reencoding, elaboration and constraint relaxation as the major mechanisms of the insight problem solving (Schooler et al., 1993; Knoblich et al., 1999). In the framework of the theory, these mechanisms operate on the mental representation alone while any motor activity is considered in its pure instrumental role as a means for expressing the solution in the physical world. The major mechanisms considered in the framework of the criterion for satisfactory progress theory, are also purely mental upon their nature. They are closely related to the solvers’ horizon of planning (lookahead) (MacGregor et al., 2001). Later, the lookahead concept has been linked to the spatial memory span (Chein et al., 2010). Note that neither of the theories predicts the change in the motor activity along the course of the insight problem solving.

One of the sources of the difficulty of the nine-dot problem traditionally considered in the literature is that during initial attempts to solve the problem the motor output is affected by irrelevant perceptual constraints imposed primarily by the square arrangement of the dots (Maier, 1930; Scheerer, 1963). We showed that successful solvers employ on-line planning for shaping their motor output and therefore that relying exclusively on the off-line planning mode is insufficient for reaching success. The relaxation of the negative impact of the perceptual grouping constraints takes place because of the influence the motor processes exert onto perceptual ones. This kind of motor-to-perception influence provides a new example of the functional role of motor activity during insight problem solving. We suggest that relying on the on-line motor planning constitute yet another possible mechanism of solving insight problems.

### Methodological Innovations of the Present Study

An attempt to study the role of motor activity in the process of solving the nine-dot problem and other insight problems faces a difficulty: a lack of dependent variables quantifying the motor activity. In order to overcome the difficulty, in the present study, we modified a traditional way of presenting the problem and scoring the solving process. In our study, participants were asked to draw line segments with the tip of the index finger on the surface of a tablet computer. The graphical movements were recorded using the specially designed custom-made software. Then, the set of recordings corresponding to multiple attempts to solve the problem were analyzed with a semi-automatic algorithm which is capable of breaking some entire recordings onto partially overlapping submovements. This allowed for separating periods of motion from the pauses between them and computing numerical estimates for movement times and pause durations. The obvious benefit of using such detailed description of solving-related motor activities is that it can be used to study the time course of the solution process.

The method that we applied allows for systematic comparison between successful vs. unsuccessful problem solvers based on the quantitative parameters of their motor activity. Using this method, we found new specific patterns of motor activity that differentiate successful and unsuccessful solvers. We hope that our approach would be helpful in further investigations of the functional role of motor activity in insight problem solving.

### Limitations

The limitations of this study include a relatively small sample size and its predominantly female composition. Besides, the study is limited to analyzing the only one problem – nine-dot problem. Another limitation of the present study was that we did not verify whether solutions demonstrated by the participants were indeed insight solutions.

### Future Directions

The proposed method makes it possible to implement several research directions. First, it seems reasonable to compare the process of solving various types of insight problems involving the motor component (for example, 6-coin, 8-coin, 6 matches etc.) from the perspective of the successful and unsuccessful solvers of the modes of motor planning. Second, a valuable contribution to understanding the mechanisms of insight problem solving would be identifying and analyzing the individual strategies in the course of solving these problems. Third, in order to uncover the details of the mechanisms of insight problem solving it worth to compare the impact of various experimental interventions (motor, oculomotor, verbal, etc.) in the form of prompting, priming or preliminary training on the process of solving insight problems involving the motor component. Finally, the mechanisms underlying the insight problem solving could be studied by comparing the parameters of motor activity shown by expert versus novice solvers. It is also interesting to compare the results obtained with the new method with the results of more traditional methods of fixating the process of solving insight problems (eye movements fixated with an eye-tracker, verbal protocols, video recording).

## Author Contributions

VS planned experiments, theoretical analysis of the results, and wrote the text of the article. NL conducted experiments, processed the data, theoretical analysis of the results, and wrote the text of the article. II conducted experiments, processed the data, and wrote the text of the article. AK processed the data, theoretical analysis of the results, and wrote the text of the article.

## Conflict of Interest Statement

The authors declare that the research was conducted in the absence of any commercial or financial relationships that could be construed as a potential conflict of interest.
